# An overview of mechanical microenvironment and mechanotransduction in intervertebral disc degeneration

**DOI:** 10.1038/s12276-025-01546-6

**Published:** 2025-10-01

**Authors:** Wencan Ke, Hanpeng Xu, Chengyi Zhang, Zhiwei Liao, Huaizhen Liang, Bide Tong, Feijun Yuan, Kun Wang, Wenbin Hua, Bingjin Wang, Cao Yang

**Affiliations:** 1 Department of Orthopaedics, Union Hospital, Tongji Medical College, Huazhong University of Science and Technology, Wuhan, China; 2 Shenzhen Huazhong University of Science and Technology Research Institute, Shenzhen, China

**Keywords:** Mechanotransduction, Ion channel signalling

## Abstract

Cellular mechanotransduction, essential for many biological functions, involves the conversion of mechanical signals into biochemical signals related to cell activities and metabolism. Physical factors in the local cellular microenvironment include external mechanical forces, mechanical stimulation generated by the extracellular matrix and intercellular mechanical interactions mediated through cell–cell adhesions. Intervertebral disc degeneration (IDD) is a complex pathological process involving diverse etiological contributors, such as mechanical wear, oxidative damage and nutritional deficiency. Notably, aberrant mechanical loading has been identified as a pivotal driver in both the initiation and progression of IDD. The mechanical microenvironment in intervertebral discs mainly includes pressure, tension, hydrostatic pressure, osmotic pressure and extracellular matrix stiffness. A thorough understanding of the mechanotransduction process of intervertebral disc cells in response to various mechanical stimuli and its regulatory mechanism is of great significance for the prevention and treatment of IDD. Here, therefore, we systematically review the research progress in understanding the mechanical microenvironment and mechanotransduction in IDD.

## Mechanotransduction insights into intervertebral disc degeneration

Low back pain is a growing global health issue, especially as the population ages. It often results from intervertebral disc degeneration (IDD), which affects the discs that cushion the spine. These discs are primarily made of a jelly-like substance called nucleus pulposus, which helps to absorb stress on the spine. Researchers explored how mechanical forces affect IDD. They reviewed studies on how different types of mechanical stress impact nucleus pulposus cells. These forces can change cell behavior and lead to disc degeneration. The study focused on mechanotransduction, which is how cells convert mechanical signals into biochemical ones. This process involves structures such as integrins and ion channels that sense mechanical changes. The researchers found that abnormal mechanical stress can lead to cell damage and disc degeneration. Understanding these processes may aid in the development of therapies to slow or reverse IDD.

This summary was initially drafted using artificial intelligence, then revised and fact-checked by the author.

## Introduction

With the acceleration of population aging, low back pain (LBP) is gradually becoming one of the main causes of global health problems, bringing great challenges to today’s aging society^1^. An analysis of the burden of disease in 195 countries shows that LBP is the leading cause of disability and workforce loss, placing a heavy economic burden on global health systems^2^. A large number of studies have shown that intervertebral disc degeneration (IDD) and its secondary pathological changes may be the main cause of LBP^3^. Intervertebral discs are avascular fibrocartilaginous structures made up of the nucleus pulposus (NP), annulus fibrosus (AF) and cartilaginous endplates, connecting adjacent vertebrae to mitigate spinal compressive forces^4^. Among them, NP tissue is the key part of intervertebral disc function and has become the focus of intervertebral-disc-related research^5^. The NP tissue is composed of extracellular matrix (ECM) and NP cells, which are jelly-like and contain large amounts of water (70–80%), so that it has the biological function of buffering the stress from different directions of the spine^6,7^. Current studies have shown that the reduction of NP cells and subsequent ECM degeneration are the main etiological and pathological mechanisms causing IDD, but these studies have not established a widely accepted disease model.

In recent years, the mechanism of mechanical microenvironment in the development of different diseases has gradually attracted the attention of researchers^8–10^. Cells are constantly subjected to external mechanical stimulation, and these mechanical signals can take various forms, including external mechanical forces, mechanical stimulation generated by the ECM and mechanical stimulation generated by the adhesion of neighboring cells. A large number of studies have shown that the biological response mediated by mechanical stimulation generated by spinal motion is an important factor in IDD. The mechanical microenvironment of NP tissues is characterized by compressive stress, shear force and matrix stiffness, which play an important role in the initiation and progression of IDD^5,11,12^. The stress on NP tissues varies dramatically with daily activities and has significant effects on NP cell function and phenotype. Previous studies have shown that abnormal stress stimulation can affect various signaling pathways in NP cells and induce autophagy and senescence in NP cells^13–16^. In addition, increased ECM stiffness during IDD further exacerbates oxidative stress and apoptosis in NP cells. Therefore, it is of great significance to explore the role and molecular mechanism of mechanical stimulation in the IDD process.

To adapt and survive, cells must respond to external mechanical stimuli. The process of mechanical signal transduction into intracellular biochemical signals is called mechanotransduction^17^. The main mechanosensors involved in these processes are integrins located in focal adhesions and cadherin found in cell–cell junctions, both linked to the actin cytoskeleton and responsible for conveying mechanical signals inside the cell^18^. For example, vascular endothelial cells can sense blood flow shear force, cause the expression of proinflammatory factors to be upregulated and, thus, regulate the biological function of vascular smooth muscle cells^19^. The stiffness of the ECM is also an important mechanical cue in regulating cell function. In liver fibrosis, sclerosing of the ECM activates the yes-associated protein (YAP) pathway in hepatic stellate cells, promoting collagen deposition and increased smooth muscle actin expression, leading to the formation of myofibroblasts^15^. In addition, the mechanical forces at cell–cell junctions are significant for tissue development and homeostasis. N-cadherin is essential in maintaining tissue integrity by regulating cell–cell adhesion between resting cells. Kruse et al. found that N-cadherin communicates through intracellular signaling and contributes to the integrity of the endothelial barrier^20^.

In this Review, we provide an overview of the mechanical microenvironment and signal pathways in the mechanotransduction of IDD based on the current knowledge and emerging research in the field. The Review is organized into three main parts. First, we briefly introduce the different types of mechanotransduction and how they influence cell fate. Then, we summarize the mechanical microenvironment in IDD and its influence on IDD progression. Finally, we review the research progress of signal pathways and molecular mechanism involved in mechanotransduction during IDD. This Review aims to enhance the understanding of the mechanical microenvironment and mechanotransduction of IDD and provide useful information for the development of therapeutic strategies to delay IDD progression.

## An overview of cell mechanotransduction

The cells live in a three-dimensional (3D) dynamic microenvironment, and its behavior is not only regulated by chemical signals, but also influenced by many mechanical signals^21^. Mechanical signals manifest in many forms, including externally applied mechanical stimuli (fluid shear, mechanical tension/pressure and ultrasound) or mechanical stimuli generated by ECM (geometry, nanomorphology and stiffness) and adjacent cells^22^. Specifically, cells sense the mechanical inputs of the microenvironment and translate them into biochemical signals that affect cell adhesion, proliferation, migration and differentiation. This mechanically related signal transduction plays a crucial part in ontogeny and homeostasis, affecting the development and progression of diseases, including muscular dystrophy, cardiomyopathy, fibrosis and cancer^23–25^.

The effect of mechanical signals on cells is bidirectional. On the one hand, mechanical stimuli are delivered via cell surface receptor (such as integrins and cadherin) as well as cytoskeleton (such as actin and microtubules) and eventually converted into biochemical signals by mechanosensitive proteins and organelles, which triggers further biological reactions^26^. Then, the perceived mechanical signals are transmitted from cytomembrane to the cytoplasm through multiple mechanisms, such as cytoskeletal remodeling. Mechanical signals can even extend to the nucleus, resulting in gene and protein expression changes, and then induce the adaptive cellular response^27^. Biochemical signals, on the other hand, regulate mechanical signals through modifications to ECM, cytoskeletal and membrane proteins^28–30^. In addition, cells are capable of producing forces that regulate the mechanical signals of surrounding cells and ECM^31,32^.

In the past few decades, researchers have made great progress in the study of molecular signaling pathways. However, the contribution of mechanical transduction in cellular responses is rarely understood and frequently neglected. Elucidating mechanotransduction mechanisms in cellular processes is therefore critical to understanding disease pathogenesis. To explore the mechanism of cell fate regulation by signal transduction, regulation of gene transcription complex and biological influence of gene itself under the stimulation of microenvironment, new clues are provided to solve the key scientific problems related to the occurrence, development, prevention and treatment of clinical diseases and to design new biomaterials.

## The type of mechanotransduction

**Fig. 1:**
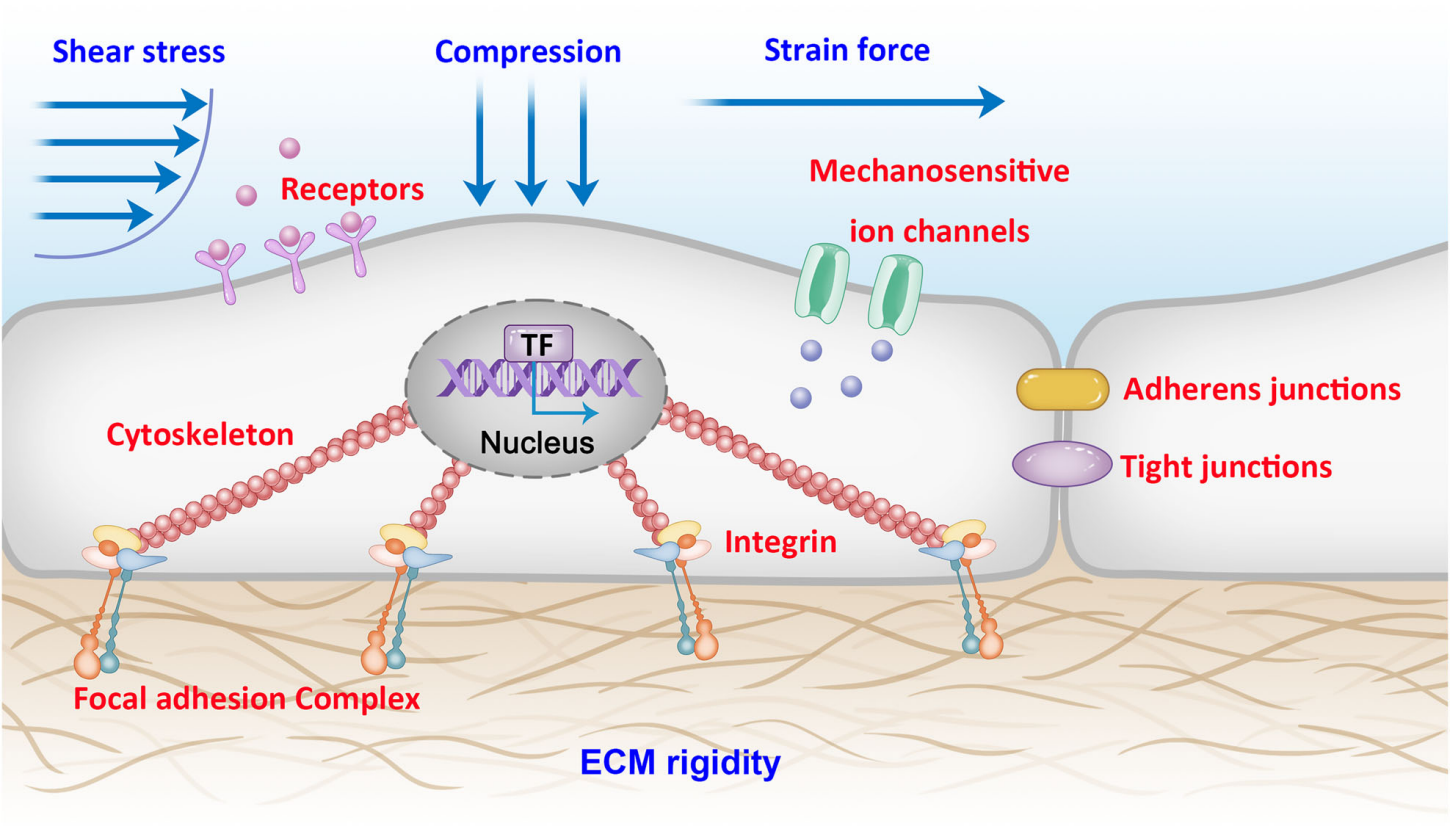
Schematic showing cell mechanotransduction along which mechanical signals transmit. Mechanical signals come in many forms, such as mechanical stimuli applied externally (fluid shear, pressure and strain force) or mechanical stimuli generated by ECM (geometry, nanomorphology and stiffness) and adjacent cells (blue text). Cells can sense these mechanical stimuli through mechanosensors such as ion channels, cell surface receptors, focal adhesions, actin cytoskeleton and cell–cell adhesions (red text).

Cells are constantly subjected to mechanical stimuli, and these mechanical signals contain many forms, including mechanical stimuli applied externally (fluid shear, tension/pressure, ultrasonic waves and so on) or generated by ECM (geometry, nanotopography, stiffness and so on) as well as mechanical stimuli generated by adjacent cells (Fig. 1). Different mechanical stimuli are sensed and delivered to cells through various signaling pathways, which together regulate the cell fate. In general, almost all cells respond to mechanical stimuli and undergo adaptive changes. These changes include both short-term responses (such as cell adhesion, diffusion or migration) and long-term effects (such as mRNA transcription, protein translation, structural recombination or cell activity).

### Mechanotransduction generated by external force

Almost all cells in the body are affected by mechanical forces, and these mechanical stimuli exert precise control over fundamental cellular behaviors, including morphogenesis, lineage commitment, intercellular communication and cell proliferation. First, these signals cause the cell membrane to deform; second, changes in membrane tension trigger responses from membrane proteins, such as mechanosensitive ion channels, and growth factor receptors^33,34^. The mechanical signals propagate through sequential biochemical events and eventually lead to complex biochemical reactions, such as modulation of the cell cycle, regulation of gene expression and induction of protein degradation or other posttranslational modifications^35^. For example, tensile force produced by blood flow induces cardiomyocyte hypertrophy and mediates the progression of cardiovascular disease^36^. In addition, fluid shear stress produced by blood flow can lead to increased stress in blood vessel walls and induce anti-inflammatory effects, such as Klf2/4 or endothelial nitric oxide synthase production^37^. In addition, chondrocytes derived from osteoarthritis cartilage may improve collagen biosynthesis through increased compressive stimulation^38^.

Mechanosensitive ion channels are expressed in various cell types and include several types, such as epithelial sodium channel (ENaC), transient receptor potential (TRP) and Piezo. The ENaC ion channel superfamily forms homotrimers or heterotrimers, with two transmembrane regions per subunit. Although the exact mechanism of mechanical gating of this ion channel family is still unclear, previous studies have found it to be sensitive to shear stress. For example, after unilateral nephrectomy, the rapid increase of glomerular filtration rate in the other kidney leads to increased fluid shear stress, which can regulate ENaC expression and reabsorption to maintain volume homeostasis^39^. The TRP protein family comprises generally nonselective ion channel proteins, with many members exhibiting mechanosensitivity, including TRPV4, TRPC1 and TRPC6^40^. TRP-mediated mechanotransduction contributes critically to cardiovascular homeostasis, pain perception, renal function and neurological regulation^41^. In 2010, Coste et al. discovered a new family of mechanosensitive ion channels in eukaryotes, including Piezo1 and Piezo 2^42^. Studies have shown that trimer Piezo ion channels are nonselective and permeable to Na^+^, K^+^ and Ca^2+^ and are involved in multiple pathological and physiological processes, including cell division, bone development and innate immunity^43–45^. The Piezo protein family can respond to various mechanical stimuli. In skin, Piezo ion channels play an important role in tactile sensing, and in endothelial cells, Piezo ion channels can respond to both shear stress and tensile stress^46,47^. In addition, growth factor receptors serve key functions in detecting and transducing biomechanical signals. Among them, transforming growth factor (TGF-β)-mediated mechanical responses play an important role in a variety of diseases, including cardiomyocytes, lung fibrosis, kidney disease, neurological disease and inflammatory bowel disease^48–50^. For example, elevated mechanical tension can activate TGF-β–Smad signaling that promotes lung fibrotic development^51^.

### Mechanotransduction at cell–ECM adhesion

The mechanotransduction at cell–ECM adhesion is mainly mediated by focal adhesion (FA), a large protein complex consisting of integrins and various linker proteins, which physically binds the ECM to the cytoskeleton and transfers mechanical signals to the nucleus^52^. Integrins are one of the most widely studied cell adhesion receptors, mediating a wide range of cell and tissue functions in tissue development and disease progression^53^. All integrins are composed of noncovalently bonded heterodimers consisting of paired α and β subunits. In mammals, 18 α subunit genes and 8 β subunit genes combinatorially generate 24 distinct α–β integrin variants^54^. Different integrin subunit combinations have different affinity for ECM components and activate different intracellular signaling pathways^55–57^. Integrins are coupled to the cytoskeleton by a large number of linker proteins, such as talin and vinculin. Talin is a key regulator of integrin activation and directly connects β-integrin to actin filaments^58,59^. Vinculin mediates the interaction between talin and actin microfilaments, modulating FA dynamics^60^. In addition, adhesion complexes also contain zyxin, paxillin, Src kinases and FAK to coordinate biochemical signaling cascades^27,61^. FA kinase or Src family proteins can mediate the diffusion of actin around the cell membrane and increase the transcriptional activity of cell proliferation, migration and differentiation^62^. Interestingly, it has been reported that integrins modulate TGF-β signaling through various mechanisms, including the regulation of pathway component expression and downstream effectors^63^.

The mechanical stimuli generated by ECM include geometry, nanomorphology and stiffness. Previous studies have shown that the topological structure of ECM has a significant effect on cell proliferation, migration and differentiation. Teo et al. found that mesenchymal stem cells form aligned stress fibers on polydimethylsiloxane nanograting with a linewidth of 250 nm and increase the expression of neurogenic and myogenic differentiation markers. The observed adhesion spots in these cells were also significantly smaller and longer on the nanograting compared with micrometer gratings or no grating^64^. In recent years, the influence of matrix stiffness on cell phenotype and function has been extensively studied. For example, the recruitment of FA complexes is largely regulated by matrix stiffness. Specifically, compared with rigid substrate, soft substrate suppresses FA complex assembly and cell expansion^65^. Matrix stiffness can also regulate the cell cycle of fibroblasts through the FA kinase pathway, as indicated by increased cyclin A expression on rigid hydrogels^66^.

### Mechanotransduction at cell–cell junctions

Cell–cell junctions are special regions on the cell membrane that consist of protein complexes that connect adjacent cells^67^. Cell–cell junctions enable tissues to resist external and internal mechanical forces while facilitating force sensing and intercellular transmission. Cell–cell junctions are specific to cell types, tissue types, developmental stages and physiological/pathological conditions and have different mechanical properties. Cell–cell adhesion complexes comprise three major forms: adherens junctions (AJ), tight junctions (TJ) and desmosomes, exhibiting distinct functional specializations^68^. Extracellular domains of cell–cell junctions interact with neighboring cells across membrane receptors, while intracellular domains interact with signaling complexes and cytoskeletons^69^. AJ are formed by interactions between the extracellular domains of type I and type II classical cadherins from neighboring cells, consisting of extracellular domains, transmembrane domains and cytoplasmic domains^70^. Cadherins are expressed in different forms in different tissues, E-cadherin and P-cadherin are widely expressed in epithelial cells, VE-cadherin expression is limited to the vascular system and N-cadherin is preferentially expressed in nonepithelial cells^71^. In addition, vinculin can also serve as mechanical sensors in AJ, although it has been studied more extensively in integrin-mediated FA. For example, the tension transmitted by VE-cadherin in endothelial cells to open the catenin protein requires the involvement of vinculin^72,73^. In addition to exchanging substances, the mechanical forces generated by cell–cell junctions also play an important role in tissue development and homeostasis^74^. For example, in epithelial tissues, mechanical signals generated by AJ play a key role in regulating basal cell differentiation, epidermal stratification and cell proliferation to maintain epithelial homeostasis^75^.

Recent studies have shown that TJ and desmosomes also act as mechanical sensors and perform biological functions during cell–cell junctions. TJ in epithelial and endothelial layers seal intercellular spaces, restricting ion and solute paracellular transport^76^. TJ are also mediated by polymeric protein complexes, including transmembrane proteins such as claudin, occludin, junctional adhesion molecules and intracellular proteins, such as zonula occluden (ZO)^69,77^. ZO proteins, including ZO-1, ZO-2 and ZO-3, are recruited into TJ by occludin, claudin and junctional adhesion molecules^78^. In addition, ZO-1 junctional recruitment involves α-catenin and vinculin associations^78,79^. In breast epithelial MCF10A cells, the absence of ZO-1 and ZO-3 led to different influence on intercellular tension, indicating TJ-mediated regulation of mechanotransduction^80^. Desmosomes mediate cell–cell junctions in the heart muscle, bladder and skin tissues, coupling with intermediate fibers to provide connection stability that preserves tissue integrity during stretching and compression stresses^81^. Desmosomes contains two subtypes, desmogleins and desmocollins^82^. Price et al. demonstrated that the force generated by the cytoskeleton had little effect on desmosomes tension, but external forces would significantly affect desmosomes tension^83^. Therefore, they believe that desmosomes are specialized for stress absorption. However, Baddam et al. found that desmosomes exhibited low-intensity tension in noncontractile cells^84^. The reasons for the above differences are not clear, but the study of desmosomes in mechanical conduction is still in the early stage. Desmosomes can interact with AJ and may influence mechanical transduction in this way.

## The mechanical microenvironment of intervertebral discs

**Fig. 2:**
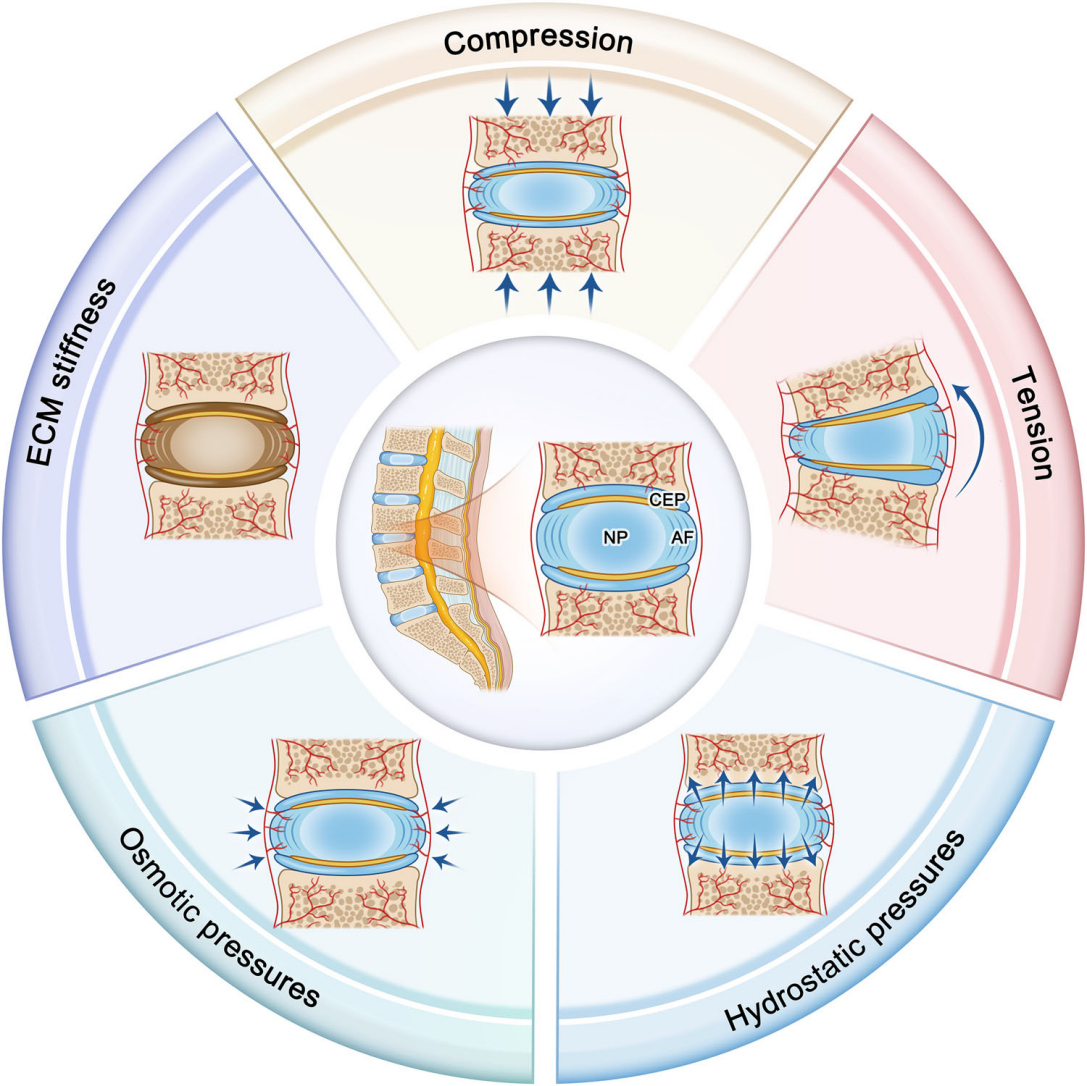
The mechanical microenvironment of intervertebral discs. The cells in the intervertebral disc are subjected to mechanical stimulation such as compression, tenure, hydrostatic pressure, osmotic pressure and matrix stiffness.

During spinal movement, the cells in the intervertebral disc are subjected to mechanical stimulation such as compression, tenure, hydrostatic pressure, osmotic pressure and matrix stiffness, which are important regulatory factors of cell activity and metabolism in the intervertebral disc (Fig. 2). Cells in different anatomical regions are exposed to a series of mechanical stimuli, and their biological response depends on where the cells are located and the intensity and frequency of the mechanical stimuli. There is growing evidence that cell-mediated remodeling of the intervertebral disc occurs under mechanical stimulation of daily activities and exacerbates the progression of disc degeneration.

### Compression

The intervertebral disc plays an important role in the bearing of compression on the spine, and the compression can vary dramatically in daily activities. Due to the inherent inhomogeneity of the intervertebral disc, the distribution of compression in NP and AF is irregular and different. Depending on external factors such as position and weight, the compression of the intervertebral disc can be divided into static compression and dynamic compression, each exerting distinct effects on the function and phenotype of intervertebral disc cells^85,86^. Previous studies have shown that intervertebral disc cells are affected by both the frequency and magnitude of compression demonstrating a threshold effect. Higher static stress (>1 MPa) caused multiple negative effects on intervertebral disc cells, including cell death, reduced expression of glycosaminoglycans and collagen and increased expression of ECM degradation proteases^87,88^. These effects may not be observed at lower compression loads (<1 MPa) or in shorter periods of time. Ohshima et al. found that low static stress (0.2–0.4 MPa) promoted anabolic responses in intervertebral disc cells, such as increased proteoglycan and collagen synthesis^89^. Studies of intervertebral disc cells responding to dynamic compression provide another example of the threshold effects of mechanical stimulation. Dynamic compression changes the cell-specific expression of ECM components (collagen I and II and proteoglycans) and matrix proteases (MMP-3, MMP-13 and ADAMTS4), which depends on the frequency and magnitude of the stress. A lower frequency (<0.5 Hz) of dynamic compression has been shown to maintain proteoglycan content and support normal intervertebral disc cell metabolism, while increased compression frequency leads to a significant increase in the number of apoptotic cells^90,91^.

### Tensile stress

During spinal activity, the size and type of mechanical stimulation have different effects in different anatomic regions, while tensile stress mainly acts on AF cells in the periphery of the intervertebral disc. Tensile stress affects proteoglycan and collagen synthesis in AF cells in a magnitude- and frequency-dependent manner^92^. Physiological tensile stress has a moderate inhibitory effect on AF cells biosynthesis, and AF cells biological response is minimal when the range is 4–6% (ref. ^93^). Studies have shown that AF cells can maintain anabolism under low intensity (1%) and physiological frequency (1 Hz) of tensile stress^94^. However, beyond this range, AF cells shift from anabolic to catabolic and lose their ability to produce matrix. In addition, high-intensity tension stimulation (5–20%) can also lead to catabolic reactions in AP cells, such as increased expression of MMP-3, COX2, NO and TNF-α, while proteoglycan production decreased^95^. The NP tissue is located inside the intervertebral disc, so NP cells are not thought to experience high tensile strain under physiological load. At 10% (0.5 Hz) or 20% (0.05 Hz) tensile stress, NP cells exhibit increased cell proliferation and collagen synthesis^96^. In addition, NP cells activate innate immune receptors such as Toll-like receptors when stimulated by short-term excessive physiological tensile stress (20%)^95^. These different responses of NP and AF cells probably reflect their adaptation to region-specific mechanical demands. NP cells experience mainly compressive and hydrostatic pressures, while AF cells are stimulated by tensile stress.

### Hydrostatic pressures

Owing to the high water content of NP tissues, NP cells are exposed to high hydrostatic pressure during both rest and activity. Hydrostatic pressure serves as an essential factor in maintaining the biomechanical stability of the intervertebral disc. The presence of hydrostatic pressure taunts the AF and supports the endplates, evenly distributing the pressure on the intervertebral disc and maintaining the intervertebral disc height. Previous studies have shown that the intervertebral disc hydrostatic pressure ranges from 0.1 to more than 3 MPa under different states^97,98^. When the hydrostatic pressure was maintained within a physiological range (0.3–1 MPa), researchers observed different phenomena. At this range of hydrostatic pressures, some studies found increased collagen synthesis, while others found increased expression of ECM degradation proteases^99–101^. This difference may be related to cell density, as there is a positive correlation between cellular confluency and hydrostatic pressure-induced stress accumulation, reflecting density-dependent mechanotransduction dynamics^102^. With aging and degeneration, the hydrostatic pressure decreased with the reduction of proteoglycan and water in NP tissues. The decrease of hydrostatic pressure leads to the reduction of intervertebral disc height and axial load bearing force, resulting in increased compression pressure on AF and facet joints. In addition, greater hydrostatic pressure (>3 MPa) leads to a degenerative response in NP cells, manifested by an increase in ECM degrading proteases and a decrease in ECM synthesis^103^. Previous studies have shown that, when the hydrostatic pressure reaches 3–10 MPa and the frequency is greater than 1 Hz, the expression of anabolic genes is downregulated and the expression of catabolic genes is upregulated in both NP and AF cells^104,105^. Neidlinger-Wilke et al. used calcium alginate hydrogel to culture NP cells in 3D environment and evaluated the effect of hydrostatic pressure on the cells^106^. They found that applying a hydrostatic pressure of 2.5 MPa at a frequency of 0.1 Hz for 30 min had no significant effect on the expression of proteoglycan, type I collagen, type II collagen, MMP-2 or MMP-3.

### Osmotic pressure

The permeable environment of the intervertebral disc is formed by a combination of mechanical deformation and fluid redistribution and can vary locally based on biochemical composition and local volume changes. Urban et al. showed that the interstitial osmotic pressure of the intervertebral disc is much higher (~430 mOsm) compared with plasma, which is related to local changes in proteoglycan density and extracellular hydration after disc compression^107^. As an important part of maintaining and regulating osmotic pressure, NP tissues are abundant in proteoglycans, especially glycosaminoglycan, allowing for tissue swelling and mechanically driven fluid flow^108,109^. This contributes significantly to the biomechanical function of the intervertebral disc, that is, the osmotic pressure of the NP tissue balances the load on the spine through its unique osmotic and water-binding properties^110^. Intervertebral disc cells exhibit the greatest rate of proteoglycan synthesis at physiological osmotic pressure (~ 430 mOsm), and either an increase or decrease in osmotic pressure has a negative effect. Previous studies have shown that changes in osmotic pressure can regulate mRNA expression of proinflammatory factor- and neurotrophic factor-related genes, but the exact mechanism by which osmotic pressure regulates intervertebral disc cell metabolism remains unclear^111,112^. Due to changes in proteoglycan and water content in aging and degraded NP tissue, the osmotic pressure of the degenerated intervertebral disc changes dramatically. A previous study explored age-related NP tissue changes under sustained osmotic loading^113^. They found that culturing NP cells under osmotic conditions that mimic a healthy intervertebral disc environment can promote ECM production and restore the mechanical function of NP tissue.

### Matrix stiffness

Under the stimulation of multiple acute and chronic risk factors, notochord-like NP cells, chondrocyte-like NP cells and fibroblast-like NP cells will be replaced successively in NP tissues. Moreover, the metabolism of ECM is also affected, such as downregulated COL2 expression and upregulated COL1 expression, resulting in increased stiffness of NP tissue^4^. Biomechanical profiling reveals a two-order-of-magnitude elevation in compressive modulus from 0.3–5 kPa (intact NP) to 20–25 kPa (degenerated state), reflecting progressive tissue stiffening during IDD pathogenesis^15,114^. The ECM serves as the primary environment for cell survival and activity, influencing cell shape, growth, differentiation and metabolism^115–117^. Bailey et al. found that increased matrix stiffness promoted nuclear translocation of myocardin-related transcription factor (MRTF) and YAP in NP cells and induced fibroblast phenotypes^15^. Previous studies of our research group showed that increased matrix stiffness activated the mechanically sensitive ion channel piezo1, resulting in increased calcium ion flow, causing senescence and apoptosis of NP cells^16^. Pharmacological intervention targeting YAP and TAZ activation (via tissue compliance maintenance) or integrin-mediated mechanosensing (through cellular signal disruption) represent emerging therapeutic strategies for decoupling biomechanical microenvironment alterations from pathological progression. Barcellona et al. found that laminin-mimicking peptide-functionalized polymer substrates could replicate the effect of soft laminin materials on cell behavior^118^. Their results show that reducing the density of laminin-mimicking peptides on a harder substrate can mimic the effect of a soft substrate on NP cells and promote the expression of a younger NP cell phenotype.

## The role of mechanotransduction in IDD

### Integrins and FA

**Fig. 3:**
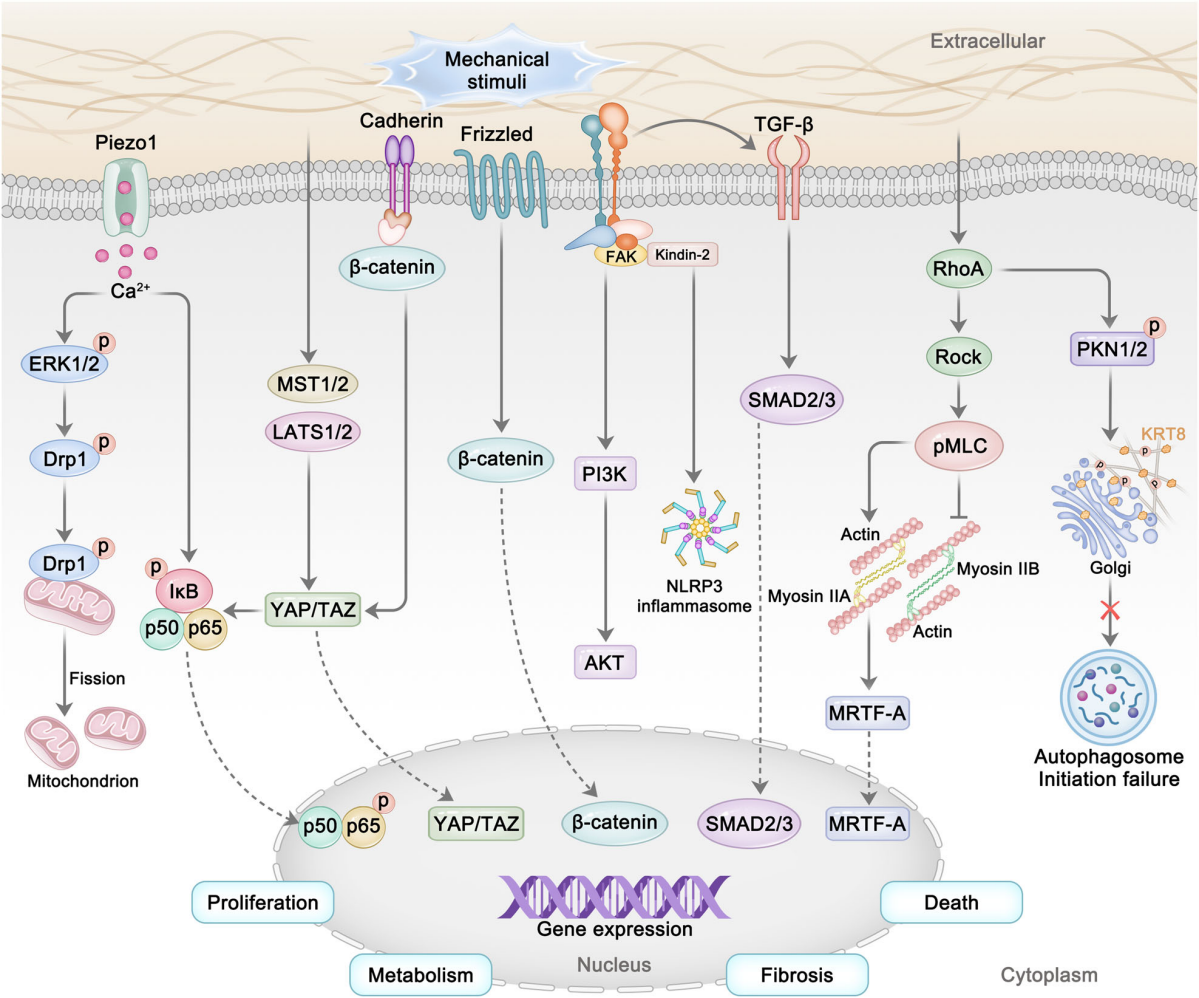
Illustration of essential molecular mechanism in mechanotransduction during IDD. Mechanosensors on the cell surface detect mechanical signals and convert them into biochemical signals, activating downstream pathways such as RhoA–Rock, SMAD2/3, Hippo–YAP, Wnt–β-catenin and others.

FA proteins, especially integrins, have been reported to play a key role in intervertebral disc homeostasis and degeneration (Fig. 3). Previous studies have shown that RGD (arginine–glycine–aspartic acid) ligands and laminin play a key role in the activation of integrin pathway in intervertebral discs. RGD, a major component of extracellular fibronectin and collagen, binds to integrin α5β1 and helps NP and AF cells respond to mechanical stimuli, such as dynamic compression and cyclic stretching. In healthy NP and AF cells, dynamic compression has been shown to reduce ADAMTS4 expression via the integrin α5β1 integrin signaling pathway. However, NP or AF cells isolated from degenerated intervertebral disc tissue were unable to reproduce this phenomenon, indicating altered intracellular mechanotransduction in degenerative condition^119,120^. Zhang et al. found that the integrin β1 signaling pathway inhibits mechanical stimulation-induced AF cells apoptosis by activating the ERK1/2 pathway under cyclic tensile strain stimulation, thus playing a protective role in responding to mechanical stimulation^121^. Wu et al. revealed that the fibronectin matrix promotes the formation of integrin-β1–syndecan-4 complex and maintains the function and survival of NP cells via the FA kinase–PI3K–AKT signaling pathway^122^. In addition, the laminin-mediated integrin signaling pathway also plays an important role in disc homeostasis. For example, laminin binds to integrins α6β4, α3β1 and α6β1 in NP cells, promotes cell adhesion and upregulates the synthesis of ECM components such as proteoglycans^123^. Further studies showed that selective laminin-mimicking peptides induced NP cells to mimic the effects of a soft substrate when culturing NP cells on a hard substrate, exhibiting higher levels of biosynthetic activity and a healthy phenotype^124^. Interestingly, Bian et al. found that mechanical stress can regulate integrin–TGF-β crosstalk, which drives the progression of IDD^125^. In this process, mechanical stress can result in excessive integrin αvβ6-mediated activation of TGF-β, leading to increased matrix proteoglycan production.

In addition to integrins, other FA proteins also play important roles in the regulation of IDD progression. Zieba et al. found that global deletion of filamin B enhanced TGF-β–BMP signaling in intervertebral discs and resulted in IDD progression during postnatal development^126^. Gao et al. studied the biological effects of periodic mechanical stress on NP cells, and the results showed that periodic mechanical stress induces ECM expression and migration in NP cells via the Src–GIT1–ERK1/2 signaling pathway^127^. In addition, results from Zhang et al. demonstrated that low-intensity pulsed ultrasound induced the upregulation of ECM expression, which was regulated by the FAK–PI3K–Akt pathway^128^. Recently, Chen et al. demonstrated that kindlin-2 deletion activates the Nlrp3 inflammasome signaling pathway, leading to spontaneous IDD-like phenotypes in the lumbar intervertebral disc and accelerating IDD progression under abnormal mechanical stress^129^.

### Cytoskeleton

The cytoskeleton, which includes actin, microtubules and intermediate filament, plays a key role in responding to mechanical stimulation^130–132^. Actin is a major cytoskeletal element that affects the stiffness of NP and AF cells, and actin integrity plays an important role in the viscoelasticity of cells^133^. In healthy NP cells, actin is distributed in the cortex of the cell periphery and throughout the cytoplasm in short punctate fibers^134^. In AF cells, the actin expression was higher and distributed throughout the cytoplasm, showing a fibroblast-like morphology. In addition, intermediate filament and microtubules are also important components of the intervertebral disc cytoskeleton, forming extensive reticular structures in the cytoplasm of NP and AF cells^135^. Intermediate filament includes six types, with the following expressed in intervertebral disc cells: types I (keratin 18/19), II (keratin 8), III (vimentin) and V (lamin). These intermediate filament subtypes have become important specific molecular markers for NP and AF cells^136^. Intermediate filament can withstand large deformations, while they are also highly sensitive to small mechanical stimuli in the extracellular environment^137^. Keratin is an important component of the cell’s response to shear loads, while vimentin is more associated with compression resistance^138,139^. The expression of actin and microtubules in intervertebral disc cells did not change significantly with age, but the intermediate filament was affected by aging. For example, the expression of keratin and vimentin in human NP cells decreases with development and maturation after birth^140^.

The cytoskeleton is in a dynamic balancing state and can be deformed and remodeled under external mechanical stimulation. Previous studies have shown that NP cells respond to osmotic pressure more slowly than AF cells, which may be due to the more dense cytoskeleton of NP cells^133^. Li et al. found that AF cells subjected to 10% cyclic strain showed increased actin and tubulin and decreased vimentin expression at both transcriptional and translational levels^93^. At the same time, the actin in AF cells formed a lot of stress fibers under the action of stretching, while microtubules and vimentin were less affected. Wang et al. found that excessive mechanical load activates RHOA–PKN and phosphorylation of Ser43 on Keratin 8, which impedes the trafficking of the Golgi-resident protein RAB33B by trapping it with Keratin 8, thereby impairing autophagosome initiation and contributing to IDD progression^141^. In addition, actin and myosin can form the actomyosin system, which together form stress fibers to produce contractile force^142,143^. The myosin family includes many types, of which myosin II is widely expressed in various tissues and cells and is involved in mechanotransduction^144,145^. Myosin II includes three isoforms, myosin IIA, myosin IIB and myosin IIC, and plays a key role in a variety of biological processes such as cell division, migration and adhesion^146–148^. Our research group found that compression stress activates the RhoA–ROCK1 pathway and then regulates the interaction of myosin IIA and IIB with actin^88^. Additional research indicated that the remodeling of the actomyosin cytoskeleton played a role in the fibrotic phenotype induced by compression stress, which is mediated by MRTF-A nuclear translocation and the suppression of proliferation in human NP cells. Another study demonstrated that MRTF-A serves as a critical regulator in intervertebral discs responding to increased ECM stiffness. The activation of MRTF-A reduces glycolysis in NP cells through a dual mechanism involving the downregulation of Kidins220 and the suppression of AMPK phosphorylation^149^. These studies highlight the mechanotransduction role of MRTF-A in maintaining NP cell metabolic homeostasis under biomechanical stress conditions, suggesting potential therapeutic targets for ECM remodeling-related IDD pathologies.

### Adherent junctions

Previous studies have shown that cell–cell junctions in intervertebral disc cells are mainly mediated by N-cadherin and E-cadherin. N-cadherin is a key marker of healthy NP cells and plays an important role in the maintenance of normal phenotype and ECM homeostasis of NP cells^150,151^. Hwang et al. found that, in the in vitro culture model of laminin functional hydrogel, only the NP cells population with high expression of N-cadherin could maintain normal morphology and phenotype, while knockdown or inhibition of N-cadherin expression would cause the loss of healthy phenotype of NP cells^152^. In addition, Li et al. found that prolonged compression stimulation led to decreased N-cadherin expression and loss of normal phenotype in NP cells, which was manifested by reduced expression of glycosaminoglycans and collagen II^153^. Niu et al. found that compression stress increase the expression of NP cell senescence-related markers (p16 and p53), while N-cadherin overexpression can reverse the senescence phenotype of NP cells and promote NP cells proliferation and ECM synthesis^154^. Recently, our study demonstrated that stiff substrate induces N-cadherin downregulation and ferroptosis in NP cells^155^. Overexpressing N-cadherin can prevent YAP from entering into the nucleus by forming a complex with β-catenin and YAP, thereby reversing ferroptosis in NP cells caused by matrix stiffness. Meanwhile, the effects of E-cadherin in response to mechanical stimulation in intervertebral disc cells have been less studied. Xu et al. found that intermittent cyclic tension can inhibit the expression of E-cadherin and increase nuclear translocation of β-catenin, thus aggravating the degeneration of cartilage endplate^156^. Overexpression of E-cadherin can inhibit the entry of β-catenin into the nucleus and restore the normal phenotype.

### Mechanosensitive ion channels

In NP cells, the mechanosensitive ion channel Piezo1 senses abnormal mechanical stress and is involved in the regulation of phenotype and function. Sun et al. demonstrated that mechanical stretch stimulation activates Piezo1 expression in NP cells, leading to increased intracellular Ca^2+^ levels and NLRP3 inflammasome activation^157^. Our research showed that Piezo1 activation plays an important role in regulating the posttranslational modifications of Drp1 and the mitochondrial fission induced by substrate stiffness^158^. Blocking Piezo1 and ERK1/2 can significantly mitigate stiffness-induced increases in ROS and apoptosis of NP cells. Another study demonstrated a self-amplifying loop of NF-kB and periostin through the Piezo1–Ca^2+^ pathway, which was involved in accelerating NP cell senescence and IDD progression^159^. According to Xiang et al., mechanical stress triggers Piezo1 activation, which is essential for regulating iron metabolism and ferroptosis in NP cells^160^. In this process, Piezo1-driven iron entry occurs independently of the transferrin receptor, and pharmacological inhibition of Piezo1 significantly reduces iron accumulation, alleviates mitochondrial ROS, and prevents ferroptosis in NP cells. In addition, TRP protein is also involved in the regulation of intervertebral disc cells in response to mechanical stimulation. It has been demonstrated that that excessive stress stimulation can activate TRPV4 protein, which mediates NP cells damage by regulating cyclooxygenase 2 and prostaglandin E2^161^. Therefore, targeted inhibition or knockout of TRPV4 may be an effective way to deal with abnormal mechanical stress-induced IDD. Easson et al. found that inhibition of TRPV4 protein under stress stimulation could reduce the release of inflammatory cytokines in intervertebral disc cells and alleviate IDD progression, but could not improve the ECM disorder in AF caused by mechanical injury^162^. Recently, a in vivo study using conditional TRPV4-knockout mice suggested that TRPV4 played an important role in regulating ECM synthesis and mediating the response of intervertebral disc tissues to mechanical stress^163^.

### Hippo–YAP and Wnt–β-catenin pathway

The Hippo–YAP pathway is a key signaling pathway in response to mechanical stimuli. YAP regulates hundreds of downstream gene pathways by binding to TEA domain family member-binding domain (TEAD) transcription factors^164–166^. Hippo signaling pathways, including Mst1/2 and Lats1/2, are the main upstream pathways regulating YAP activity^165,167,168^. Activated Mst1/2 and Lats1/2 can directly phosphorylate YAP, allowing YAP to locate in the cytoplasm and inhibit transcriptional activity. When the Hippo signaling pathway is inhibited, YAP dephosphorylates and enters the nucleus, co-activating TEAD and downstream gene targets. YAP can sense a wide range of mechanical signals, including shear stress, cell shape and ECM stiffness, and translate them into cell-specific transcriptional programs. For example, when cells sense low-level mechanical signaling, YAP is mainly located in the cytoplasm, such as round cells attached to soft ECMs. By contrast, YAP is mainly localized in the nucleus when cells sense high-level mechanical signaling, such as cells cultured on rigid substrates or undergoing deformation^169,170^. In intervertebral discs, elevated matrix stiffness has been reported to stimulate aberrant proliferation of fibroblast-like NP cells via the YAP–Cyclin B1 axis, while inhibition of YAP by verteporfin can suppress this proliferation and alleviate IDD^171^. In addition, Wang et al. found that applying a 5% cyclic tensile strain reduced inflammation and encouraged the growth of AF cells by preventing YAP phosphorylation and NF-κB nuclear localization, while the application of 12% cyclic tensile strain notably induced a proinflammatory response by turning off YAP activity and turning on NF-κB signaling^172^. Recently, research showed that the Hippo signaling pathway constitutes a critical regulatory mechanism in cartilage endplate remodeling triggered by lumbar spinal instability. Mechanistically, activation of Hippo signaling or targeted knockout of its core effector gene Yap1 within endplate chondrocytes effectively attenuates IDD following lumbar spinal instability surgery. Transcriptomic sequencing further identifies chemokine CCL3 as a mechanosensitive factor abundantly released by stress-exposed chondrocytes, which drives osteoclast recruitment and formation for cartilage endplate remodeling^173^.

The Wnt–β-catenin pathway is crucial for controlling the development of various tissues and organs and is often linked to mechanotransduction. In the absence of Wnt ligands, β-catenin is phosphorylated by GSK3 and directed toward ubiquitin-dependent breakdown^174,175^. However, in the presence of a Wnt ligand, which inhibits GSK3 activity, β-catenin accumulates and translocates to the nucleus and transactivates target gene promoters^176^. It has been reported that intermittent cyclic mechanical tension exacerbates endplate chondrocyte degeneration via the Wnt–β-catenin signaling pathway^177^. According to Lu et al., β-catenin is crucial for spine tissue stability, and its abnormal elevation causes severe degeneration of the spine^178^. In addition, β-catenin has been found to be form a transcription complex with YAP and translocate into the nucleus, increase NP cells apoptosis and ECM decomposition, and accelerate the progression of IDD^179^.

## Conclusions and perspectives

Intervertebral disc cells are subjected to different types of mechanical stimulation during spinal movement, which serve as key regulators of cell activity and metabolism in the intervertebral disc. Cells in different anatomical regions of the intervertebral disc experience diverse mechanical stimuli, and their biological response depends on their anatomical location and the intensity and frequency of the mechanical stimulation. Mechanical stimuli are delivered into the cells via cell surface receptors (such as integrin and cadherin) and mechanosensitive ion channels (such as Piezo1 and the TRP proteins), which then transmit mechanical signals to the nucleus via the cytoskeleton and mechanosensitive proteins to regulate the function and phenotype of NP and AF cells. Previous studies have observed differences in the response of NP and AF cells to mechanical stimulation, and this difference is related to the regulatory mechanism of cytoskeletal remodeling.

In the process of reviewing the literature, we found that there are still some shortcomings in the study of mechanotransduction in IDD. For example, intervertebral disc cells are subjected to multiple mechanical stimuli at the same time, but current studies often use only one mechanical stimulation; therefore, subsequent studies need to explore whether there is an interaction between them. In addition, current studies on the role of integrins in the intervertebral disc cells focus mainly on integrin α5β1; future studies need to more comprehensively verify the exact role of different ligand and integrin interactions, as well as the influence of ligand density and class on downstream cell signaling pathways. The role of cell–cell junctions in maintaining intervertebral disc homeostasis has been preliminarily verified, and the specific mechanisms of cell–cell junctions in regulating phenotype and function of AF and NP cells need to be further explored in the future. Moreover, the role of mechanosensitive ion channels in NP and AF cells in response to mechanical stimulation is still limited, focusing mainly on Piezo1 and TRP proteins. Further investigation of the links between mechanosensitive ion channels and downstream signaling pathways will enhance our understanding of the physiological functions of intervertebral disc cells. The cytoskeleton consists of multiple components, and current research has focused primarily on actin, while the role of microtubules in the IDD process remains to be further explored. In addition, existing studies on mechanical stimulation in IDD are predominantly conducted in 2D models, which cannot fully replicate the in vivo environment. Future studies are recommended to further validate and investigate these mechanisms in 3D models.

In summary, mechanobiology studies of intervertebral disc cells in healthy and degenerative states may uncover new potential targets to mitigate or reverse the effects of abnormal biomechanical stimuli on intervertebral disc cells. These findings will expand our understanding of the mechanical microenvironment and mechanotransduction of intervertebral discs, advancing the development of therapeutic strategies for treating IDD.
